# Scholarly research productivity is not related to higher three-year licensure pass rates for physical therapy academic programs

**DOI:** 10.1186/s12909-015-0431-1

**Published:** 2015-09-11

**Authors:** Chad E. Cook, Michel D. Landry, Jeffrey Kyle Covington, Christine McCallum, Chalee Engelhard

**Affiliations:** 1 Doctor of Physical Therapy Division, Department of Orthopedics, Duke University, 2200 W. Main St. Ste B230, Durham, NC 27705 USA; 2 Walsh University, 2020 East Maple Street, North Canton, OH 44720 USA; 3 Department of Rehabilitation Sciences, College of Allied Health Sciences, University of Cincinnati, 3202 Eden Avenue, Cincinnati, OH 45267-0394 USA

## Abstract

**Background:**

In the domain of academia, the *scholarship of research* may include, but not limited to, peer-reviewed publications, presentations, or grant submissions. Programmatic research productivity is one of many measures of academic program reputation and ranking. Another measure or tool for quantifying learning success among physical therapists education programs in the USA is 100 % three year pass rates of graduates on the standardized National Physical Therapy Examination (NPTE). In this study, we endeavored to determine if there was an association between research productivity through artifacts and 100 % three year pass rates on the NPTE.

**Methods:**

This observational study involved using pre-approved database exploration representing all accredited programs in the USA who graduated physical therapists during 2009, 2010 and 2011. Descriptive variables captured included raw research productivity artifacts such as peer reviewed publications and books, number of professional presentations, number of scholarly submissions, total grant dollars, and numbers of grants submitted. Descriptive statistics and comparisons (using chi square and t-tests) among program characteristics and research artifacts were calculated. Univariate logistic regression analyses, with appropriate control variables were used to determine associations between research artifacts and 100 % pass rates.

**Results:**

Number of scholarly artifacts submitted, faculty with grants, and grant proposals submitted were significantly higher in programs with 100 % three year pass rates. However, after controlling for program characteristics such as grade point average, diversity percentage of cohort, public/private institution, and number of faculty, there were no significant associations between scholarly artifacts and 100 % three year pass rates.

**Conclusions:**

Factors outside of research artifacts are likely better predictors for passing the NPTE.

## Background

In the domain of academia, the *scholarship of research* (hereby described as research) may take the form of publications, presentations, or grant submissions. At the individual academician level, research has been a valuable mechanism for activities such as promotion and tenure [1]. Institutionally, research is associated with higher university rankings within healthcare programs for academic institutions [2]. Further, research has been a mechanism for development of future leaders [3] and has been used to promote new knowledge and growth in clinical practice [4].

Within the profession of physical therapy, research productivity has long been an interest among academic faculty, academic administrators, and leaders within the profession [5]. Like most health professions, the bulk of research productivity is produced in academic settings, although the productivity varies notably across academic physical therapist programs. With respect to peer reviewed publications, a 2004 study [6] reported that approximately 13 % of academic physical therapists programs reported no research artifacts, whereas 50.3 % of the programs had fewer than 5 citations. Conversely, 3 % of academic physical therapists programs had 44 or more citations, and the majority of the very productive programs were housed in Carnegie classifications of doctoral intensive or extensive institutions.

In the United States of America (USA), a physical therapist’s entry-level education has evolved over time, and is now a clinical doctorate degree (Doctor of Physical Therapy-DPT degree). At present, there are over 200 accredited programs in a multitude of different types of universities that offer a physical therapist doctoral degree. Although programs within the disparate universities differ in Carnegie classification, focus, and size, all are expected to meet the accreditation requirements that are currently governed by the *Commission on Accreditation in Physical Therapy Education* (CAPTE) [7].

In order to legally practice physical therapy in the United States, one must first graduate from a CAPTE accredited physical therapy (PT) program, and then pass the National Physical Therapist Examination (NPTE) [7]. The NPTE is administered by *the Federation of State Boards of Physical Therapy* (FSBPT), and is a standardized written examination. The FSBPT posts three year average pass rates for each CAPTE accredited program. The pass rate for the licensure exam is considered a quantifiable marker of success for an academic program. This assumption has prompted a number of studies [8–18] to evaluate predictive factors of pass rates on NPTE such as grade point average (GPA), class size, and cohort characteristics to programmatic pass rates.

As stated, research productivity has long been an interest (and expectation) of academic faculty, academic administrators, and leaders of the profession [5]. While it may be intuitive to assume that an environment that fosters research activities and promotes a culture of discovery could offer unique learning experiences for students that are immersed within the program of study, to our knowledge this has not been reported in the literature. Theoretically, those involved in research could reasonably transfer new knowledge to students, which could improve their capacity as a clinician. Furthermore, the knowledge gained by students who are in scholarly-rich environments potentially could have enhanced learning success as measured by the NPTE exam as one of the exam’s primary roles is to insure those who pass it have the knowledge needed to obtain licensure to practice in the physical therapy field [7]. Quantification of research artifacts may include publications, presentations, grants, and other works such as books or book chapters [19, 20], and all of these measures are values captured by CAPTE. Since three year FSBPT pass rates are a quantitative measure that is standardized among all academic physical therapist programs in the USA, we endeavored to determine if there were associations in all forms research artifacts (e.g., publications, grants, etc.) and with higher/lower NPTE pass rates. We hypothesized that programs with higher reported scholarly artifacts would also have higher pass rates.

## Methods

### Design

This observational study involved administrative database exploration. Data for this study were obtained by request from CAPTE and represented all accredited PT programs from the USA who graduated physical therapy students in 2011. CAPTE requires each accredited program to submit an *Annual Accreditation Report* (AAR) and the report is a comprehensive document that includes information about curriculum models, finances, format, admissions, and enrollment. In addition, all CAPTE AAR reports require programmatic graduate rates, outcome data and programmatic faculty information, including scholarly accomplishments. Data are reported annually by each program by either the program director or a designee.

Data associated from the three year NPTE pass rates were provided indirectly by the FSBPT. CAPTE worked with FSBPT to embed the three year NPTE pass rates within the CAPTE AAR dataset in a de-identified fashion. The data from this study were received in June of 2014 and included the most recently tabulated results from the CAPTE AAR. The study concept was approved and expedited by the Institutional Review Board of Duke University, Durham, North Carolina; USA (protocol ID, Pro00056918).

### Dataset characteristics

CAPTE AAR data included full datasets from 2009, 2010, and 2011. Our focus was on the reported CAPTE AAR data from the 193 accredited programs in 2011. For this study, data from programs were included if; 1) three-year NPTE pass rates were recorded, 2) programmatic resource data were included within the CAPTE AAR, and 3) a cohort graduated in 2011. A total of 8 programs were involved in a transition year (masters to a doctorate degree) and did not graduate a cohort in 2011.

We selected the cohort of 2011 because it is the most recently available cohort with full three year pass rates. However, it is important to recognize that the three year program pass rates also contain data from 2009 and 2010 cohorts. FSBPT could not provide separately reported three year pass rates for 2011 cohort only. Consequently, the 2011 cohort data in this study functions as a proxy for the full 2009–2011 cohorts. We elected not to combine the 2009–2011 cohort metrics because of the dynamic nature of faculty composition in most physical therapy programs.

### Variables used in the modeling

Variables captured included both descriptive and research-oriented values. Descriptive variables included public or private status, Carnegie status (Bachelors, Masters, Doctoral, Research, and Special Focus), geographic region (East North Central, East South Central, Middle Atlantic, Mountain, New England, Pacific, South Atlantic, West North Central, and West South Central), parent school description (Allied Health Sciences Center, Liberal Arts, Osteopathic, Professional, and Technical), public or private status, school size by student population (extra-large, large, medium, small, and extra-small), average age of the program student, average size of the graduating cohort, undergraduate cumulative grade point average, number of reported core programmatic faculty, and programmatic racial diversity. School size was divided into five categories based on enrollment of students in the parent institution. Extra-large included schools with >20,001 students, large included students with 10,001 to 20,000 students, medium included students with 4001 to 10,000 students, small included students ranging from 1001–4000 and extra small involved schools with 1000 or fewer students. All values were taken directly from the CAPTE AAR with the exception of programmatic diversity which was calculated by taking the total of non-white students divided by the total cohort, multiplied by 100.

Research artifacts included; a) peer reviewed publications (total number reported in 2011), b) total number of published books reported in 2011, c) total number of professional presentations reported in 2011, d) total number scholarly artifacts submitted but not yet published reported in 2011, e) total number of faculty with grants reported in 2011, f) summative total of grants dollars reported in 2011, and g) total numbers of grants submitted in 2011.

Within the dataset, three-year FSBPT pass rates are provided as percentage scores. The percentage scores are heavily negatively skewed with many programs reporting a three year FSBPT pass rate of 100 %. We dichotomized each into; 1) 100 % three year pass rate and 2) <100 % three year pass rate.

### Control variables

A number of variables could potentially influence three year licensure pass rates thus we elected to control for any variables that were significantly different (between programs with and without 100 % 3 year pass rates) when examined in the descriptive characteristics. Further, because we examined raw research artifacts, we realized that a larger faculty volume may increase the total number of artifacts purely by number. Lastly, although the Graduate Record Examination (GRE) was recognized as a predictor of pass rate in previous studies [10, 13, 14] and was a variable within the CAPTE AAR dataset, within the dataset GRE was inconsistently (verbal, quantitative, or both) and infrequently reported (<50 % of cases). Because of this we did not select this variable for investigation.

### Data analysis

All analyses were performed using Statistical Package for the Social Sciences, version 22.0 (SPSS Inc, Chicago, Illinois). Comparative analyses of institutional characteristics, including means and standard deviations and frequencies with percentages, were reported for Carnegie status, public/private status, region, institution type, and institution size for the 185 included programs within the CAPTE AAR. Comparative analyses among the seven research artifact variables were performed as well, with all instances using chi square, Fisher exact, or t-tests as appropriate.

Univariate logistic regression analyses, adjusted for covariates that were significantly different among programs with and without 100 % pass rates were performed for each of the independent variables for three-year pass rates. For each univariate analysis, individual *P* values, odds ratios and 95 % confidence intervals, and Nagelkerke values were reported. For each analysis, we included the control variables of public/private status, GPA, program cohort diversity, and number faculty. A Nagelkerke is a pseudo R square measure that investigates the usefulness of the model [21]. The value is similar in concept to the coefficient of determination (R^2^) in linear regression. The R^2^ statistics do not measure the goodness of fit of the model but indicate how useful the explanatory variables are in predicting the response variable and can be referred to as measures of effect size.

## Results

The cohort consisted of 185 different physical therapy programs with full licensure pass rate and scholarship productivity measures. Significant differences were present among those with and without 100 % pass rates in public or private status (public universities had higher frequencies of 100 % pass rates), grade point average (programs with higher pass rates had higher GPAs) and diversity in student cohort race (programs with 100 % pass rates had less diverse cohorts) (Table 1).

**Table 1:** Descriptive Statistics of the Program Characteristics Categorized by Mean Pass Rates (*N* = 185)

Variable	Full Sample	100 % 3 Year Federation Pass Rate	<100 % Federation Pass Rate	*P* value
	Mean (SD)/Frequency (*N* = 185)	Mean (SD)/Frequency (*N* = 107)	Mean (SD)/Frequency (*N* = 78)	
Carnegie Status	4 = Bachelors	1 = Bachelors	3 = Bachelors	0.06
	76 = Masters	25 = Masters	51 = Masters	
	20 = Doctoral	12 = Doctoral	8 = Doctoral	
	59 = Research	29 = Research	30 = Research	
	26 = Special Focus	11 = Special Focus	15 = Special Focus	
Geographic Region	30 = East North Central	16 = East North Central	14 = East North Central	0.23
	10 = East South Central	4 = East South Central	6 = East South Central	
	38 = Middle Atlantic	10 = Middle Atlantic	28 = Middle Atlantic	
	9 = Mountain	4 = Mountain	5 = Mountain	
	14 = New England	5 = New England	9 = New England	
	16 = Pacific	10 = Pacific	6 = Pacific	
	31 = South Atlantic	16 = South Atlantic	15 = South Atlantic	
	21 = West North Central	8 = West North Central	13 = West North Central	
	16 = West South Central	5 = West South Central	11 = West South Central	
Public or Private Status	86 = Private	28 = Private	58 = Private	0.01
	99 = Public	50 = Public	49 = Public	
University Type	63 = AHSC	33 = AHSC	30 = AHSC	0.16
	114 = LA	43 = LA	71 = LA	
	3 = Osteopathic	0 = Osteopathic	3 = Osteopathic	
	4 = Professional	2 = Professional	2 = Professional	
	1 = Technical	0 = Technical	1 = Technical	
University Size	31 = X-Large	19 = X-Large	12 = X-Large	0.12
	42 = Large	17 = Large	25 = Large	
	38 = Medium	17 = Medium	21 = Medium	
	65 = Small	21 = Small	44 = Small	
	9 = X-Small	4 = X-Small	5 = X-Small	
Cohort Size	41.94 (15.68)	40.62 (15.52)	42.91 (15.80)	0.33
GPA of Program	3.48 (0.30)	3.57 (0.14)	3.42 (0.36)	<0.01
Average Age of Class	24.02 (1.63)	23.95 (1.34)	24.06 (1.82)	0.67
Racial Diversity of Program (%)	16.12 (17.60)	12.93 (12.47)	18.44 (20.29)	0.04
Number of Programmatic Faculty	10.72 (4.18)	11.20 (4.82)	10.37 (3.83)	0.19

*AHSC* Allied Health Science Centre, *LA* Liberal Arts, *GPA* grade point average, *X-large* Extra large, *X-small* Extra small

Significant differences in programs with and without 100 % three year pass rates were found in the research productivity artifacts of; 1) scholarly artifacts submitted (*p* = 0.01), 2) number of faculty with grants (*p* = 0.04), and 3) grant proposals submitted (*p* < 0.01). In each case, higher amounts of artifacts were found in programs with 100 % pass rates (Table 2).

**Table 2:** Comparative Analyses of Raw Reported Scholarship Achievement Variables

Variable	100 % Federation Pass Rate	<100 % 3 year Federation Pass Rate	*P* value
	Mean (SD)/Frequency	Mean (SD)/Frequency	
Peer review publications	13.18 (14.06)	10.13 (12.31)	0.12
Number of Books	2.42 (2.83)	1.71 (2.32)	0.07
Number of Presentations	26.65 (22.11)	22.37 (21.65)	0.19
Scholarly Artifacts Submitted (but not published)	11.60 (11.14)	8.10 (7.69)	0.01
Number of Faculty with Grants	3.78 (2.95)	2.94 (2.53)	0.04
Grants Dollars Reported	$2,328,609.48 ($6,014,545.47)	$1,124,597.67 ($4,476,812.79)	0.12
Grants Proposals Submitted	3.12 (2.66)	2.00 (2.35)	<0.01

**Table 3:** Adjusted Univariate Logistic Regression Analyses examining Associations between Reported Scholarship Achievement Variables and 3 Year Federation Pass Rates. Dependent Variable is 3 Year Pass Rate (1 = Above Median, 2 = Equal or Below Median). Covariate Controls include a) Mean undergraduate GPA, b) Number of full and part time faculty, c) Public or Private status, and e) Program cohort diversity

Variable	Odds Ratio (95 % Confidence Interval)	*P* value	Nagelkerke R^2^
Peer review publications	1.00 (0.97, 1.03)	0.83	0.02
Number of Books	1.06 (0.93, 1.21)	0.38	0.02
Number of Presentations	0.99 (0.98, 1.02)	0.91	0.01
Scholarly Artifacts Submitted	1.03 (0.99, 1.07)	0.17	0.05
Number of Faculty with Grants	1.07 (0.92, 1.24)	0.38	0.03
Grants Dollars Reported	1.00 (1.00, 1.00)	0.28	0.02
Grants Proposals Submitted	1.17 (0.99, 1.39)	0.07	0.07

When univariate logistic regression modeling was used while controlling for public/private status, GPA, program cohort diversity, and number faculty, we found no significant associations between any of the research productivity artifacts and whether or not a program had a 100 % three year pass rate. In all occasions, the Nagelkerke R^2^ yielded very small values suggesting nominal influence toward three year pass rates (Table 3).

## Discussion

The purpose of this study was to determine if there were associations between all forms of research artifacts (e.g., publications, grants, etc.) and higher/lower NPTE pass rates. Because exposure to research could theoretically create an environmental culture that is more conducive to discovery, we hypothesized that programs with higher three year pass rates were likely also to have higher reported rates of scholarly artifacts. Descriptively, in all scholarship categories (peer reviewed publications, books, etc.) programs with higher pass rates reported more numerous scholarly artifacts. However, in the adjusted univariate regression analyses, there were no associations between scholarly artifacts and three year pass rates. A plausible explanation is that the pass rates may be influenced more by what faculty are teaching and students learn, regardless of scholarship production by faculty.

To explain further, when covariate controls that reflected the culture and constitution of academic institution in which the physical therapist program was housed were used in the univariate regression modeling, the total productivity differences were not statistically significant. This finding outlines the considerable interaction between research productivity and one’s academic setting of employment. Within regression analyses, an interaction reflects a confounding issue [22], a situation where one predictor variable (research artifacts) is associated with both the outcome variable (higher or lower three-year FSBPT pass rates) and another independent variable (geographic region, public or private status, grade point average, or university type). It is quite possible that a program’s scholarship is reflective of the expectations, culture and research foci of the particular institutional setting [23], just as it is possible that these components may have influenced the unadjusted comparative findings.

Keeping in mind that the DPT is an entry level, clinical doctorate degree, not a research-intensive degree, the particular culture and research foci of an institution can also influence the level at which a student is involved in research be it projects, presentations, or simply becoming informed consumers of research. Additionally, students’ interactions with faculty members who perform research can significantly vary as faculty who teach in physical therapy curriculums may have divided responsibilities. Some faculty have primary responsibilities that are in research and as a result have reduced teaching loads where other faculty have primary responsibilities that are teaching and often have reduced research expectations. Therefore, the institutional setting and the characteristics of that environment are what influence three-year FSBPT licensure rates. These characteristics were not captured within our study but we feel that they could potentially be related to resources, learning opportunities, programmatic expectations, and other non-research related endeavors.

For research productivity to truly influence teaching one would have to assume that research and teaching are complementary roles and activities, justifying and enhancing the other [24]. Further, an assumption would need to be made that the knowledge gained by the learner could theoretically improve his or her ability to pass a licensure examination, especially if the research has a direct influence on clinical practice. As previously stated, we know of no physical therapy educational studies that have explored this concept, although several non-clinical, education-based studies have been performed. Early studies (pre-1985) investigating research productivity and university faculty teaching effectiveness have shown little empirical support [25]. Feldman [26] suggested only a very small relationship between the two outputs. A follow up meta-analysis, performed 11 years later that consisted of a synthesis of 58 non-clinical studies demonstrated no association between research productivity and teaching effectiveness [27]. Webster [25] argues that the two concepts (teaching and research productivity) are truly different dimensions and do not complement one another.

Health profession licensure examinations such as the NPTE measure entry-level to practice preparation by assessing the lowest level of acceptable clinical competency. This is evident in that fact that almost all physical therapist education programs in the USA had close to a 100 % pass rate in the three year pass rate analyses. In fact, the pass rate data were heavily skewed toward 100 %, so profoundly that log linear adjustment resulted in no improvements. We feel that this descriptive finding and the underlying objective of the NPTE should be considered when looking at our result. By nature of its purpose, the NPTE does not aim to assess top performance, but rather it aims to assess a threshold of acceptability to begin practice. Thus, it is highly likely that the NPTE does not distinguish program excellence; although it may have the capacity to distinguish a few select programs who do not meet acceptable competencies.

### Limitations

Although also a mechanism beyond our control, it is possible the accuracy of reporting by each academic program is variable, especially when report of grant dollars was used. The CAPTE AAR does provide an operational definition for each question but interpretation is always a risk associated with required programmatic survey estimates.

## Conclusions

Although research productivity was associated with higher and lower pass rates, other covariates incorporated within the regression model notably adjusted the influence. This finding demonstrates the importance of comprehensive statistical modelling when analyzing different factors in educational programs but also suggests an influence of the institution that one learns within.

## Abbreviations

AAR: Annual accreditation report
ANCOVA: Analysis of covariance
CAPTE: Commission on accreditation in physical therapy education
FSBPT: Federation of State Boards of Physical Therapy
GPA: Grade point average
NPTE: National physical therapy examination
PT: Physical therapy
